# The plant holobiont: integrating molecular priming and ecological legacies for climate-adaptive immunity

**DOI:** 10.1042/EBC20250056

**Published:** 2026-07-30

**Authors:** Raquel Campos-Herrera, Sergio Alvarez-Ortega, Victoria Pastor

**Affiliations:** 1 Department of Viticulture, Institute of Vine and Wine Sciences (ICVV: University of La Rioja, CSIC, Government of La Rioja), 26007, Logroño, La Rioja, Spain; 2 Department of Biology and Geology, Inorganic Physics and Chemistry, Rey Juan Carlos University, Móstoles Campus, 28933, Móstoles, Madrid, Spain; 3 Department of Biology, Biochemistry and Natural Sciences, Jaume I University, BIO4STRESS, Associated Unit to ICVV-CSIC, Castellón de la Plana, 12071, Spain

**Keywords:** ecology, holobiont, host-microbe Interactions, priming stimulus, root exudates

## Abstract

Plants serve as central hubs of complex network connecting above- and below-ground inhabitants. These relationships are further shaped by abiotic factors that impact the performance of all organisms in direct or indirect contact. Plant immunity orchestrates the outcomes of these interactions through multiple layers of perception, signal integration, and chemical responses. Although biotic and abiotic dynamics are highly visible in the phyllosphere, the soil represents a vast interface of constant interaction, including the effects of abiotic stressors. As a core component of plant immunity, contact with soil organisms contributes to the complex architecture of plant defense, leveraging the second functional genome to bolster an extended plant immune response. Consequently, continuous contact with organisms will serve as a priming stimulus, fostering systemic resilience against future challenges, particularly in a landscape where environmental fluctuations directly modulate pathogen virulence and soil health. In light of recent literature, the present review calls for the integration of ecological contexts into molecular studies of plant immunity, bridging the gap between cellular mechanisms and ecological dynamics to address the challenges of climate uncertainty.

## Introduction

Plants are directly exposed to environmental fluctuations, relying on extensive physiological and molecular plasticity for survival [1]. Throughout the day, they must adjust their metabolic and developmental programs in response to day–night fluctuations in temperature, light, and humidity. These adjustments involve precise regulation of photosynthesis, transpiration, and gene expression through highly integrated signaling networks. Additionally, seasonal transitions and episodic climatic extremes impose further selective pressure on plant species. While seasonal changes can be perceived by plants as signals to synchronize their development, adverse environmental episodes activate complex stress response pathways, ranging from large-scale transcriptional and metabolic adjustments to preserve cellular homeostasis [2]. In the environment, not only do abiotic factors influence plant physiology, but continuous contact with soil biota (i.e. bacteria, fungi, nematodes, arthropods, earthworms) also shapes the reprogramming of plant physiology and immunity [3]. Interestingly, beneficial soil organisms can enhance plant performance by promoting growth, strengthening defense responses and increasing tolerance to environmental disturbances, such as frost, floodings, droughts, or heat waves [4]. However, abiotic factors can affect soil health and biodiversity [5,6], making it essential to consider soil biodiversity, ecosystem multifunctionality, and plant–soil interactions to understand system responses in each scenario [7]. Despite significant progress in plant immunity, microbiome research, and soil ecology, a critical knowledge gap remains: we still lack an integrated framework that explains how plants coordinate defense responses within the broader phytobiome under fluctuating environmental conditions. Although current studies often examine abiotic stress responses alongside microbial interactions or focus on soil biodiversity in isolation [5,8], they frequently overlook how these components interact to collectively shape plant resilience, particularly under climate-driven disturbances [5,7]. This gap constrains our ability to effectively harness soil multifunctionality and beneficial biotic partnerships to improve stress tolerance and advance sustainable crop protection. In the present review, we synthesize recent advances across five interconnected thematic areas. First (i), we examine defense priming and its influence in the architecture of plant immunity. Next (ii), we focus on the rhizosphere-immune interface, exploring how plants perceive soil biota, regulate root barriers, and integrate systemic signals into immune responses. We then (iii) discuss the plant holobiont as a multitrophic network in which soil organisms actively contribute to plant defense and ecological legacies. Subsequently (iv), we consider how environmental change modulates plant–organism interactions and shapes the expression of the extended plant immune system. Finally (v), we outline future challenges and opportunities for integrating soil biodiversity, plant immunity, and climate resilience into a holistic framework for sustainable crop production. By bridging mechanistic and ecological perspectives across these interconnected levels, the present review frames plant immunity as an extended phenotype, or extended plant immune system, emerging from dynamic interactions between the host, its microbiome, and the surrounding environment that collectively contribute to plant defense beyond the host genome alone.

## Defense priming and the architecture of plant immunity

The plant immune system allows plants to adapt to biotic stresses through a complex network of perception and signal transduction events [9]. Constitutive barriers (like phytoanticipins, cell wall, cytoskeleton, cuticle, or trichomes) are the first layer biotic threats can encounter when landing on the plant surface, since they are always present in plants. As a second barrier, inducible defenses are displayed when a biotic threat (pathogen or/and pest) attacks the plant. But some of these inducible defenses may be boosted after a primary contact of plant tissue with a biotic or non-biotic stressor, producing a transient and slight plant response. Such responses can be expressed as direct local or systemic defenses [10]. Alternatively, they can initiate priming, a state that enhances the plant’s defensive phenotype without immediate activation. Afterwards, when a biostressor appears, stimulated plants will activate their responses more efficiently and will overcome the infection faster [11–13]. This phenomenon is called defense priming. The priming stimulus produces cellular responses by different molecular mechanisms, which depend on the priming state. Three main states are considered: (a) the priming state, triggered by a priming stimulus; (b) the post-challenge primed state, which occurs when the stressor starts colonization and infection; and (c) transgenerational primed state, which is found in the offspring of damaged plants [14]. The cellular responses triggered during the priming phase can confer both short-term and long-lasting defensive capacity. Specifically, molecular mechanisms underlying this stress memory encompass a broad spectrum of metabolic reconfigurations, ranging from alterations in cellular redox homeostasis to stable epigenetic modifications [15,16]. Bridging cellular mechanisms with an ecological context requires viewing plants as components of the broader phytobiome, which integrates the plant host, its associated biological communities, the surrounding environment that modulates their interactions, and the dynamic network of processes connecting them [17]. Priming stimuli can originate from multiple biotic sources and reach the plant through direct contact, such as in the rhizosphere, rhizoplane, endosphere, or phyllosphere, or indirectly via bulk-soil organisms or endophytes residing in other hosts. The persistence and functional outcomes of priming are highly context-dependent, shaped by environmental conditions, microbial identity, and the physiological status of the plant [7,18,19].

## Rhizosphereimmune interface: integrating local microbiota perception into the plant’s extended immune phenotype

### Perception of soil-borne microbes

Different players orchestrate the interaction between plants and microbes. The rhizosphere serves as the active interface for root–microbe interactions, where its composition is governed by the host genotype and critically influences plant fitness [20]. Immune activation is primarily triggered by the perception of microbe- or pathogen-associated molecular patterns (MAMPs or PAMPs, respectively). This recognition initiates microbe- or pathogen-triggered immunity (MTI or PTI, respectively), allowing the plant to gate microbial entry [21]. Although pathogens may bypass MTI to facilitate infection, plants remain calibrated in their response by integrating microbial presence with endogenous danger signals (DAMPs). This contextualization ensures that defense mechanisms are proportionate to the threat, mitigating the fitness costs associated with excessive resource allocation [21,22]. For example, in the model Arabidopsis–*Pseudomonas simiae* WCS417 interaction (a classical plant growth-promoting rhizomicrobe

(PGPR)), the perception of the flagelling22 *flg22^417^* epitope could initiate PTI, and it is observed that when bacterial cells are alive, this potential immune activation is actively quenched to maintain the ‘sound of silence’ [23], a state of immunological homeostasis where the beneficial microbe (*Pseudomonas simiae* WCS417) actively suppresses local host defenses to facilitate stable colonization while mitigating the metabolic fitness cost of immune activation. This suppression is primarily driven by bacterial gluconic acid biosynthesis, which modulates extracellular pH to stifle early PTI signaling events and reactive oxygen species (ROS) production [24]. Such molecular negotiations are estimated to occur in approximately 40% of rhizosphere bacteria, allowing the plant to bypass costly growth–defense trade-offs, effectively integrating beneficial partners into its extended immune system while remaining primed for genuine pathogenic threats [23].

Complementing this bacterial strategy, the plant host prevents chronic overstimulation by spatially restricting the expression of pattern recognition receptor (PRRs) such as *FLAGELLIN SENSITIVE2* (*FLS2*), to specific cell layers [25]. For example, it has been demonstrated that in *Arabidopsis*-differentiated root tissues the expression of PRRs is low; consequently, PTI is not activated upon treatment of the PAMP *flg22*, even at high concentrations of 1 μM. However, when the same spatial root cells perceive both MAMPs and DAMPs from neighboring cells, the receptor-like kinase *FRK2* is induced, thereby triggering MTI-related defenses. In contrast, in outer tissues of the root tip, *flg22* can initiate immune responses of varying intensities across different concentrations without the requirement of damage [21]. Interestingly, since root-colonizing microbes primarily inhabit the elongation and transition zones, the occurrence of cell damage serves as a critical checkpoint to differentiate the plant’s response, allowing it to distinguish between ‘friend’ and ‘foe’ [21,26]. On the other hand, in leaf tissue, plants invest energy in distinguishing between MAMPs and DAMPs, but when detecting both, the defenses are boosted in a faster and stronger manner. This effect was demonstrated *in vitro* experiments: when co-applying the DAMPs cADP or cellobiose, and the PAMP *flg22* plants boosted the MTI by increasing the levels of phytocytokines, ROS, and genes relative to PTI [22]. Moreover, when applying the same DAMPs separately prior to infection with foliar pathogenic *Pseudomonas syringae* pv *tomato* DC 3000 they were able to induce resistance in tomato plants [22]. Interestingly, the co-application restored the susceptibility to the level of control plants, pointing to the specificity of DAMP perception and signaling.

### Physical and chemical plant barriers in roots

Beyond microbial perception, root structural barriers and exudates play central roles in recruiting, shaping, and modulating plant-associated microbiota [19,23,27]. Physical barriers such as the epidermis, cortex, and endodermis regulate microbial access by integrating cues from both microbial identity and the presence of cellular damage. Endodermal specializations, including the Casparian strip and suberin lamellae, function as selective gateways that control metabolite flux between roots and soil, thereby influencing the assembly and composition of the root microbiome [19,28–30].

The diffusion of metabolites into the soil can be called exudates if the compounds are water soluble, or rhizodeposits, consisting in other plant metabolites, plant debris, or root cap cells [31]. Among the most described exudates can be considered organic acids, amino acids, flavonoids, sugars, vitamins, and phenolics [23,32], which can serve as carbon sources and nutrients for the microbes present in the soil, thereby contributing to shape the microbial community in the rhizosphere [30,33]. The chemical profile of exudates is fundamentally a product of plant genotype, although it is significantly modulated by the environment. The synergy between abiotic conditions (e.g. pH and soil structure) and biotic factors (micro- and macroorganisms) results in a dynamic composition that shifts according to external pressures [34]. Despite the fluctuations in exudate chemistry, it is important to consider that the metabolic functions of the soil microbiome often remain stable due to functional redundancy. As Louca et al. [35] demonstrated, while environmental and host-derived factors drive significant turnover in specific microbial taxa, the essential biogeochemical functions are maintained by diverse but functionally overlapping communities. This stable functional recruitment constitutes a key component of the plant’s extended phenotype, shifting the focus of plant defense from ‘who’ is present to ‘what’ functions are performed to ensure host fitness.

Beyond these primary and secondary metabolites, the plant employs a sophisticated specialized metabolism to filter its environment. For instance, *Arabidopsis thaliana* utilizes biosynthetic gene clusters to produce triterpenes such as talianin and arabidin. These compounds do not function as broad spectrum antibiotics but as selective modulators that actively shape the root microbiota by promoting or inhibiting specific microbial taxa according to plant’s requirements [36]. This genotypic control of the chemical profile is further refined by a stringent ‘economic’ framework to manage mutualism. Plants can distinguish between high-quality partners and cheaters through two-step process. During the initial ‘molecular handshake’ plants use lysine motive LysM-type receptors to perceive symbiotic signals like Nodulation (Nod) factors or mycorrhizal lipochitin oligosaccharides (Myc-LCOs), ensuring entry only to compatible partners. Once the symbiosis is established, the plant monitors nutrient exchange [37]. For instance, if a microsymbiont fails to deliver adequate N or P, the host imposes post-colonization sanctions such as triggering arbuscule collapse in mycorrhizae or restricting oxygen in rhizobial nodules, effectively cutting off the carbon supply to unproductive guests [33].

### Systemic signals in plant defense

Beyond the physical and chemical properties of roots in contact with soil and its biota, systemic signal transmission from roots to shoots (or vice versa) represents an additional layer of plant interaction. For example, it is well established that microbial contact can trigger specific forms of induced resistance (IR), classified as SAR or ISR depending on the stimulus and the systemic signals involved. Classically, SAR is associated with initial contact by necrotizing pathogens, utilizing local salicylic acid (SA) responses and emitting systemic signals such as N-hydroxypipecolic acid (NHP), methyl salicylate, and azelaic acid among others [37–39]. Following the perception of these mobile signals, SA is synthetized *de novo* in distal leaves, at intermediate concentrations compared with infected tissues. This moderate amount of SA accumulation can activate the SAR master regulator NPR1, by disrupting the NPR1–NPR4 interaction, preventing NPR1 degradation, and facilitating its monomeric accumulation of NPR1 in the nucleus, priming the plant for further attacks [40]. This process is regulated by a pivotal TGA- and NPR1-dependent transcriptional module that facilitates the induction of SAR and ensuring a rapid and potent induction of systemic defense genes upon subsequent challenges [41,42].

On the other hand, ISR describes IR phenotypes elicited by beneficial soil organisms or other stimuli (e.g. chemicals), involving systemic signals and may engage defensive pathways beyond the classical jasmonic acid/ethylene and SA [12,42–44]. Interestingly, root-derived NHP (classical signal for SAR) serves as a systemic layer of microbial discrimination, between beneficial and pathogenic interactions. According to Xu et al. [45], roots maintain an NHP ‘standby circuit’, controlled by the balance between Flavin-containing monooxygenase 1 (FMO1)-mediated synthesis and UDP glucosyltransferase76B1 (UGT76B1)-mediated inactivation, that functions as a biotic sensor. The differential intensity of this NHP signal, specifically modulated by the nature of the microbial encounter, allows the plant to strategically evaluate the trade-off between growth promotion and the activation of systemic immunity. This ability to dynamically calibrate defense responses, together with stable functional recruitment, based on root-to-shoot signaling, constitute key components of the plant’s extended phenotype, ensuring host fitness in complex environments [45]. Other systemic signals derived from plant–microbe interaction is described in mycorrhiza-induced resistance. Different signals have been detected in the tomato xylem and transported from root to shoots such as yatein and secoisolariciresinol, independently of the infection, but with higher accumulation in the roots and xylem of mycorrhizal tomato plants. Interestingly, yatein shows antifungal effects, and its accumulation in leaves is higher upon infection of the pathogen *Botrytis cinerea* [46].

## The plant holobiont: orchestrating multitrophic defenses and ecological legacies

**Figure 1:**
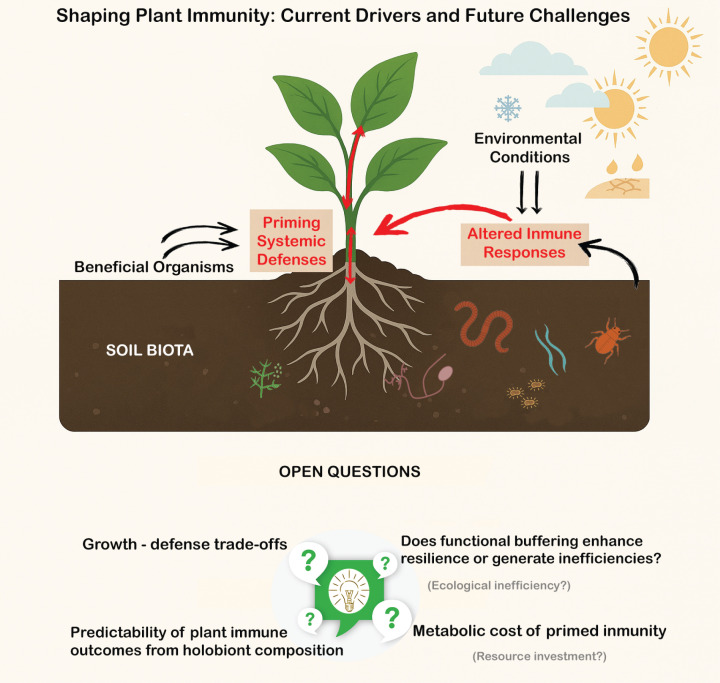
The extended plant immune system: drivers, modulators, and future perspectives The diagram illustrates the central role of the plant–soil system within the holobiont framework. Elements shown in black represent the biotic and abiotic factors shaping plant immunity, whereas elements in red indicate the resulting biological consequences or outcomes. Black arrows denote the influence or impact of a given factor on a biological process, while red arrows represent the resulting response or consequence. Beneficial organisms, including microorganisms and soil fauna (such as PGPRs, beneficial fungi, microarthropods, earthworms, or entomopathogenic nematodes) contribute to plant defense by promoting induced systemic resistance and defense priming. Simultaneously, environmental conditions can modify belowground trophic networks, alter ecological interactions among soil organisms, and directly affect plant physiology, thus influencing immune responses. Together, these biotic and abiotic drivers shape the expression of the extended plant immune system and determine plant resilience under changing environmental conditions. The figure also highlights key knowledge gaps and future research challenges, including growth–defense trade-offs, metabolic costs associated with maintaining a primed state, whether functional redundancy within soil-biota communities enhances resilience through functional buffering or instead generates ecological inefficiencies, and the predictability of plant immune outcomes from holobiont composition and ecological context. Addressing these questions will be essential for developing biodiversity-based strategies and climate-resilient cropping systems.

The modern understanding of plant immunity has shifted from a host-centric model towards an ‘extended phenotype’ framework [17], where the plant holobiont functions as a coordinated defensive unit. This integration is rooted in a sophisticated chemical dialogue within the rhizosphere and on the phyllosphere (Figure 1). Contact with microbes reprogram plant cells enhancing resistance, through modulation of signaling and metabolic pathways, in what is called ‘cry-for-help’ strategy [47] in plant exudome or in cell reprogramming. In addition to antimicrobial compounds, plants exudate signal molecules and nutrients that shape the rhizosphere microbiome. Certain microbes may then establish endophytic populations, colonizing the phyllosphere as well, eventually reaching the seeds for vertical transmission to the subsequent generation [48,49].

Evidence demonstrates that pathogens can modify the root exudome and plant defenses to attract beneficial organisms [20,31,50]. It was observed that *Stenotrophomonas rhizophila* (SR80) accumulated in the roots of wheat plants following infection with the pathogenic fungus *Fusarium pseudograminearum*. Moreover, when re-introducing the bacterium into the soil, the plants exhibited protection against the same pathogen [51]. Additionally, *Hyaloperonospora arabidopsidis* (*Hpa*)-infected plant leaves of *Arabidopsis*, assembled a core-community of 25 protective taxa, named *Hpa*-associated microbiota, in the phyllosphere although transmitted to the soil creating a soilborne legacy, suppressing disease in future plant generations [20]. Furthermore, Xu et al. [52] demonstrated that *Pseudomonas* species accumulate in wheat heads during *Fusarium graminearum* infection. These beneficial microbes secrete organic acids to reduce the pH, serving as a counterstrategy against the fungus, which relies on alkalinization to facilitate the disease. In general, the orchestration of such beneficial microbiomes requires strict immunological regulation, as demonstrated by Pfeilmeier et al. [53,54]. This work shows the relevance of nicotinamide adenine dinucleotide phosphate reduced (NADPH) oxidase respiratory burst oxidase homologue D (RBHOD) in suppressing the pathogenic activity of resident/opportunistic *Xanthomonas* strains, which can deploy its type II secretion system (T2SS) to trigger dysbiosis in *Arabidopsis* plants. Strikingly, while the host represses this genus aboveground, it is recruited belowground during pathogen attack jointly with other taxa, initiating ISR [55]. Collectively, these studies underline the complex immune network where host survival relies on suppressing opportunistic *Xanthomonas* in the leaves while recruiting the counterparts in the soil in a cry-for-help strategy.

Recent evidence further indicates that this plant–soil interaction can also operate in the opposite direction, whereby soil biota release chemical cues that plants perceive and use to activate their immune responses. For instance, earthworms and their cutaneous excreta prime tomato plants by enhancing JA-mediated defenses and sustaining the upregulation of antifungal genes such as *LOX-D*, *PIN-II*, and *GH-19*, thereby increasing resistance and improving biological control of *B. cinerea* [56]. Moreover, earthworms can reshape soil bacterial communities and enhance the activity of other biocontrol agents that protect plants against root herbivores [57], underscoring the complexity of soil-mediated regulation of plant immunity. Similarly, in potato, belowground beneficial organisms such as arbuscular mycorrhizal fungi and entomopathogenic nematodes can modulate plant immunity by altering constitutive phenolics and reshaping volatile organic compound (VOC) emissions in the leaves, responses that can be quantitatively assessed through phenolic profiling and VOC analysis [58]. Together, these findings highlight that root–shoot associated physical and chemical barriers are not static structures but dynamic interfaces capable of integrating signals from diverse soil organisms, ultimately shaping the plant’s systemic immune landscape

Beyond microbially mediated root ‘cry-for-help’ responses, plants can also recruit higher trophic levels as part of their extended defense strategy. Plants under herbivore attack can emit specific VOCs that recruit protective mesofauna, notably entomopathogenic nematodes that kill root-feeding insects [59,60]. Subsequent studies have revealed additional complexity in this interaction: for instance, Ali et al. [61] demonstrated that the same plant cues can also attract natural enemies of these nematodes, such as nematophagous fungi, potentially undermining the defensive benefit of the signal. Likewise, Chiriboga et al. [62] showed that colonization of maize roots by *Pseudomonas* spp. enhances herbivore-induced immunity by increasing the production of the sesquiterpene (E)-β-caryophyllene and upregulating its synthase gene tps23 during rootworm attack, thereby strengthening indirect defenses by improving the plant’s ability to attract entomopathogenic nematodes. These multitrophic interactions highlight that plant defense is not restricted to direct antimicrobial activity but extends to the ecological recruitment of beneficial organisms across trophic levels. Together, these findings underscore that soil biota act not merely as passive associates but as integral components of an extended plant immune system, reshaping defensive signaling networks and ultimately redefining how plants perceive, process, and respond to biotic threats.

## Immunity in a changing climate: environmental modulation of the holobiont’s extended phenotype

Instead of examining plant responses to individual abiotic stresses, we focus here on how environmental conditions reshape plant–biota interactions and their contribution to the extended plant immune system. Climate change is altering the ecological and molecular context that governs plant–microbe interactions [63]. Climate extremes such as heatwaves, droughts, and intense rainfall can suppress plant immune pathways, increase pathogen virulence, and destabilize microbiome functions, thereby shifting disease outcomes in favor of the pathogen [64–66]. These disruptions extend to systemic immune regulation: fluctuating temperatures and water availability influence the NHP-dependent signaling circuit that underpins SAR, altering the balance between NHP synthesis and inactivation and affecting defense calibration under stress [67–69].

Environmental fluctuations also reshape root exudation, modifying the release of organic acids, amino acids, flavonoids, and specialized metabolites that structure microbial recruitment in the rhizosphere [70,71]. Such shifts can enhance colonization by beneficial microbes or open niches for opportunistic pathogens, depending on the type and intensity of stress [71]. At the same time, stress legacy effects-persistent changes in plant physiology and microbiome composition following prior drought or heat, can influence how future stresses are perceived and mitigated, either stabilizing mutualisms or exacerbating maladaptive plant–microbe feedback. In addition, recent studies have shown that beneficial microbes contribute key buffering mechanisms under climate stress, including 1-amincyclopropane-1-carboxylate (ACC)-deaminase-mediated ethylene regulation, ROS detoxification, improved osmotic adjustment, and enhanced nutrient mobilization [72,73]. These microbial functions are increasingly conceptualized as part of a ‘second functional genome’ expanding its adaptive capacity beyond its own genetic repertoire [74]. Understanding how environmental variability modulates this extended functional system is therefore essential to advance towards sustaining plant resilience under rapidly fluctuating environmental conditions. Together, these findings show that climate change modulates plant–microbe interactions at multiple levels (from molecular immune signaling to rhizosphere assembly and long-term ecological feedback), highlighting the need to integrate microbiome-based strategies into climate-adaptive agriculture.

## Future research and perspectives

Understanding plant–biotic interactions under fluctuating environmental conditions requires moving beyond single-organism and single-scale perspectives. Plant immunity and resilience emerge from coordinated processes within a dynamic soil–plant–microbe–fauna continuum, highlighting the need to study these interactions in real soils where microbial communities, soil fauna, and roots respond collectively to stress (Figure 1). Within this context, the integration of diverse beneficial soil organisms remains understudied and their deployment in microbial consortia requires systemic evaluation of compatibility and complementary functions across different soils and cropping systems.

Progress will further rely on integrated multi-omics approaches capable of linking molecular reprogramming to soil biodiversity, food-web structure, and ecosystem functions, enabling the identification of resilience markers and clarifying how beneficial soil organisms influence plant stress tolerance. As climate change intensifies environmental and pathogen pressures, it becomes essential to understand how soil biota buffer plants and how stress legacies shape present plant–soil feedback. A major challenge ahead is understanding how plants balance the metabolic costs of immune vigilance with growth and reproduction under simultaneous abiotic and biotic stresses. Likewise determining whether microbiome and faunal functional redundancy promotes resilience through functional buffering, thereby stabilizing community functions under recurrent stress, or instead generates ecological inefficiencies remains an important open question (Figure 1).

Ultimately, translating this knowledge into practice will require system-level models that connect belowground diversity, soil functions, and plant defense outcomes, providing predictive frameworks to guide biodiversity-based strategies that strengthen soil health, reduce reliance on chemical inputs, and support sustainable crop production under future environmental scenarios. This insight allows us to design smarter, diversity-driven support the rational design of biotic-informed agricultural practices, including the use of beneficial microbial and faunal consortia, the promotion of soil biodiversity and the optimization of defense priming strategies to enhance crop resilience under increasingly variable environmental conditions.

## Glossary

PGPR: Beneficial soil-borne bacteria that colonize the rhizosphere and enhance plant fitness and defense.

MAMPs/PAMPs: Highly conserved pieces of molecular signatures characteristic of microbes/pathogens.

DAMP: Endogenous molecules released from the plant cell into the apoplast, including cell wall fragments or cytoplasmic debris, which can be recognized by extracellular receptors in the neighboring cells. They act as a danger signals, primary if the fragments are directly released or secondary (or phytocytokines) if they are previously processed after damage.

PTI/MTI: First layer of plant immune response when a pathogen/microbe is perceived.

PRRs: Cell surface receptor that recognizes molecular patterns from self (DAMPs) or non-self (DAMPs or PAMPs) interactors.

Flg22: A highly conserved 22-amino acid peptide derived from the N-terminus of bacterial flagellin. Natural variations in its sequence (epitopes) such as those found in specific beneficial or symbiotic bacteria, can differentially modulate host immune receptor activation, allowing plants to distinguish between mutualistic ‘friends’ and pathogenic ‘enemies’.

Nod factors/Myc-LCOs: Class of specialized molecules secreted by beneficial microorganisms (rhizobia and arbuscular mycorrhizal fungi, respectively) to initiate mutualistic symbioses with plant roots.

Extended plant immune system/extended plant immunity: Conceptual framework describing plant immunity as an emergent property of the entire holobiont. It is shaped by the dynamic interplay between the plant, its microbiota, soil biota, and environmental drivers. Under this model, immune competence extends far beyond the plant’s own genome, actively incorporating functional traits and ecological safeguards provided by its associated microbial and faunal communities.

## Summary

- Plant immunity should be considered as holobiont, functioning as an extended phenotype that actively integrates the diverse biological and abiotic elements of the surrounding ecosystem
- Contact with beneficial organisms serves as a natural priming stimulus that potentiates inducible defenses. This primed state enhances immune responsiveness upon pathogen attack while helping balance resource allocation between defense and plant development.
- The precise perception of the micro/macro biota is critical for plant fitness. To distinguish beneficial from pathogenic traits, plants must engage in complex molecular ‘negotiations’ and reprogramming their immune signaling to mount a context-specific response.
- The root–soil interface acts as the central hub for ecological recruitment. Integrating local biota perception with dynamic exudation, roots orchestrate the assembly of beneficial belowground communities and initiate long-distance signaling to prime systemic immunity.
- To face the challenges of climate, future research must bridge the gap between host-centered molecular mechanisms, and the ecological dynamics of the plant extended immune system.

## Open Access

Open access for this article is provided via our Subscribe to Open programme, made possible through the support of our subscribing institutions. Learn more here: https://portlandpress.com/pages/open_access_options_and_prices#conditional.

## Abbreviations

flg22: flagelling22
IR: induced resistance
LysM-type receptors: lysine–motiv
MAMP: microbe-associated molecular pattern
MTI: microbe-triggered immunity
Myc-LCOs: mycorrhizal lipochitin oligosaccharides
NHP: N-hydroxypipecolic acid
Nod factors: nodulation factors
NPR1/NPR4: non-expresser of pathogenesis-related genes 1 and 4
PAMP: pathogen-associated molecular pattern
PGPR: plant growth-promoting rhizobacteria
PTI: pathogen-triggered immunity
PRR: pattern recognition receptor
ROS: reactive oxygen species
SA: salicylic acid
VOC: volatile organic compound
DAMP: damage-associated molecular pattern
ISR: induced systemic resistance
SAR: systemic acquired resistance
